# Sestrin2 in diabetes and diabetic complications

**DOI:** 10.3389/fendo.2023.1274686

**Published:** 2023-10-18

**Authors:** Xiaodan Zhang, Zirui Luo, Jiahong Li, Yaxuan Lin, Yu Li, Wangen Li

**Affiliations:** ^1^ Department of Endocrinology, The Second Affiliated Hospital of Guangzhou Medical University, Guangzhou, China; ^2^ The Second Clinical Medicine School, Guangzhou Medical University, Guangzhou, China

**Keywords:** diabetes, diabetic complications, Sestrin2, oxidative stress, mitochondrial function

## Abstract

Diabetes is a global health problem which is accompanied with multi-systemic complications. It is of great significance to elucidate the pathogenesis and to identify novel therapies of diabetes and diabetic complications. Sestrin2, a stress-inducible protein, is primarily involved in cellular responses to various stresses. It plays critical roles in regulating a series of cellular events, such as oxidative stress, mitochondrial function and endoplasmic reticulum stress. Researches investigating the correlations between Sestrin2, diabetes and diabetic complications are increasing in recent years. This review incorporates recent findings, demonstrates the diverse functions and regulating mechanisms of Sestrin2, and discusses the potential roles of Sestrin2 in the pathogenesis of diabetes and diabetic complications, hoping to highlight a promising therapeutic direction.

## 1 Introduction

Diabetic mellitus (DM) is a metabolic disease characterized by hyperglycemia. It is a type of disease in which defects in insulin production and activity lead to abnormal glucose metabolism. Continuous hyperglycemia leads to impaired cellular autophagy and oxidative stress response, which further induces inflammatory response and the stimulation of coagulation, and finally gives rise to the occurrence of complications in multiple organs and systems (1). In recent years, the number of patients with diabetes has increased dramatically globally due to an aging population, changes in the lifestyle, and the increase prevalence of obesity. Among various types of DM, type 2 DM (T2DM) is the major type and accounts for nearly 90% of all DM cases. Diabetes and diabetic chronic complications have become important causes of disability and death for individuals, and have posed huge economic burdens worldwide (2).

As an important member of the Sestrins (Sesns) protein family, Sestrin2 is a newly discovered stress-inducible protein widely distributed in animals. Sestrin2 gene was originally identified in human neuroblastoma cells as a hypoxia-activated gene (3). Sestrin2 accumulates in mammalian cells in various pathophysiological states such as hypoxia, starvation, radiation, oxidative stress and endoplasmic reticulum (ER) stress (4). Previous studies on Sestrin2 mostly focused on metabolic disease such as obesity, age-related diseases and malignant tumors. Recent researches indicated that Sestrin2 also plays critical roles in the pathogenesis of cardiovascular diseases, kidney diseases, liver diseases, respiratory diseases, and diseases of the nervous system and exerts protective effects on several organs (5). Complicated mechanism is involved, including regulation of oxidative stress, mitochondrial function, ER stress, autophagy, metabolism and inflammatory response (5, 6). In recent years, increasing numbers of studies report about correlations between Sestrin2 and diabetes, indicating that Sestrin2 might become a novel therapeutic target for diabetes. In this review, we summarize the recent findings and discuss the potential role and underlying mechanism of Sestrin2 in diabetes and diabetic complications.

## 2 Sestrin2 pathways and modulating mechanisms

As a sensitive stress receptor, Sestrin2 is activated in stress conditions. A variety of adverse stresses could promote Sestrin2 expression, such as oxidative stress, ER stress, hypoxia, energetic stress, and age- and obesity-associated metabolic pathological conditions (7–10). Up-regulated Sestrin2 exerts pleiotropic biological effects in diverse physiological and pathological states, through attenuating oxidative stress, and modulating a series of cellular events such as autophagy, ER stress, mitochondrial biogenesis, protein synthesis, cell energy homeostasis and apoptosis, while many of these responsive pathways are interconnected (5, 11–14).

### 2.1 Upstream factors of Sestrin2 signaling

In response to stress, Sestrin2 could be regulated by various transcription factors, including tumor-suppressor protein p53, hypoxia inducible factor-1α (HIF-1α), nuclear factor erythroid 2-related factor-2 (Nrf2), nuclear factor-κB (NF-κB), activated transcription factor 4 (ATF4), c-Jun NH(2)- terminal kinase (JNK)/c-Jun, Foxhead box O3 (FoxO), activated protein 1 (AP-1), and CCAAT. A series of stress conditions, such as oxidative stress, ER stress, DNA damage, hypoxia and mitochondrial dysfunction, provoke the release of these upstream factors, and result in altered expression and activity of Sestrin2 (15, 16). The mediating effects of Sestrin2 in stress conditions will be further discussed below.

### 2.2 Downstream pathways of Sestrin2 signaling

After being induced, Sestrin2 thereafter mediates several signaling pathways, including adenosine monophosphate-activated protein kinase/mammalian target of rapamycin (AMPK/mTOR) pathway (17), Kelch-like ECH-associated protein1/Nrf2 (Keap1/Nrf2) pathway (18), the mitogen-activated protein kinase8/JNK1 (MAPK8/JNK1) pathway (19), AMPK/peroxisome proliferator-activated receptor γ coactivator-1α (AMPK/PGC-1α) pathway (20), extracellular regulated protein kinases (ERK1/2) pathway (21), thrombospondin-1/transforming growth factor-β1/Smad3 (TSP-1/TGF-β1/Smad3) pathway (22), and TGF-β/NADPH Oxidase 4 (NOX4)/ROS signaling pathway (23). Among these signaling pathways, AMPK/mTOR and Nrf2/Keap1 are the principal ones that Sestrin2 is suggested to be involved in the pathogenesis of diabetes and diabetic complications.

#### 2.2.1 The AMPK/mTOR pathway

Comprising of two distinct protein complexes (mTORC1 and mTORC2), mTOR functions as a crucial sensor for energy, nutrient, and redox states, and thereafter regulates protein synthesis and autophagy (24). Persistent mTOR stimulation is linked with a wide range of diseases such as diabetes, obesity, cardiovascular diseases, cancer, autoimmune diseases and metabolic disorders (25). mTORC1 can phosphorylate and suppress autophagy-initiating protein kinases unc-51-like kinase 1 (ULK1), so as to inhibit cellular autophagic catabolism (26). mTORC2 is insensitive to nutrients but is sensitive to growth factors via phosphatidylinositol 3 kinase (PI3K) signaling, and thereby regulates metabolism and cytoskeletal tissue (27) and functions as a crucial controller of lipid metabolism (28). mTORC1 could negatively regulate mTORC2 activity. mTOR is strongly associated with T2DM and many of its chronic complications. Both mTORC1 and mTORC2 play significant roles in the regulation of insulin signaling. mTORC1/ribosomal protein S6 kinase 1 (S6K1) and mTORC2/protein kinase B (AKT), is critical for the maintenance of insulin sensitivity and that their dysfunction contributes to the development of T2DM (29). mTORC1 in pancreatic β-cells controls cell size, proliferation, survival, maturation, protein translation, insulin processing and secretion, and autophagy. mTORC1/S6K1 pathway regulates the apoptosis and autophagy of β-cell, while mTORC1/4E-BP2-eIF4E pathway regulates the proliferation of β-cell. Loss of β-cell-specific mTORC1 leads to diabetes and β-cell failure (30). mTORC2 is essential for maintaining a balance between the proliferation and the cell size of β-cells (31). Recently, it is reported that mTORC2 regulates glucose-stimulated insulin secretion in β-cell by enhancing actin filament remodeling (32). Besides the direct regulating effects on β-cell, mTORC2 also modulates glucose uptake in peripheral tissues including adipose, skeletal muscle and brown adipose tissues (33–35). Moreover, mTORC2 participates in the regulation of hepatic insulin sensitivity, glycolysis, and lipogenesis (35, 36).

Sestrin2 exerts antioxidant and apoptosis-associated effects in a variety of diseases through the inhibition of mammalian target of rapamycin complex 1 (mTORC1) and/or the activation of AMPK (21–23). Sestrin2 can suppress mTORC1 through AMPK-dependent or -independent pathways. It was presumed that Sestrin2 regulates AMPK activation by orchestrating the recruitment of liver kinase B1 (LKB1), independent of calmodulin-dependent protein kinase 2 (CAMKK2), as well as promoting LKB1/AMPKα1β1γ1 complex expression (37, 38). Except the AMPK signaling pathway, Sestrin2 can also directly bind to RagA/B regulatory protein complex 2 (GATOR2), mediate the discharge of GATOR1, stimulate GATOR1 to inhibit RagA/B, and suppress mTORC1 inimitably (39). Sestrin2 promotes mTORC2 activity through its interaction with mTORC2, as well as the inhibition of feedback loop (40). GATOR2-mTORC2 axis is essential for Sestrin2-induced AKT activation (41), which exerts various glucose- and lipid-regulating effects (42).

#### 2.2.2 The Keap1/Nrf2 pathway

As a member of a family of basic leucine transcription factors, Nrf2 is involved in a serious of important cellular events including redox regulation, DNA repair, metabolic homeostasis, and apoptosis prevention (43). Nrf2 acts as a crucial transcription factor that can modulate antioxidant gene expression through its interaction with the antioxidant response elements (AREs). Keap1 acts as a sensor of oxidative stress, as well as a inhibitor of Nrf2 (44). Under physiological circumstance, Keap1 binds to Nrf2 in the cytoplasm and is inactivated (45). Keap1/Nrf2 signaling plays a key role in diverse diseases, including diabetes, cancer, neurodegenerative diseases, airway diseases, inflammatory diseases, cardiovascular diseases, and aging (44, 46, 47). A growing body of evidence suggest Nrf2 as a key regulator in the development and progression of diabetes and its complications (43). Nrf2 contributes to the suppression of inflammation of pancreatic β-cell, the maintenance of autophagy in pancreatic β-cells under ROS stimulation, and the regulation of the ER-associated degradation (48, 49). Besides regulating β-cell function, Keap1/Nrf2 pathway also demonstrates protective effects in diabetic complications, i.e. diabetic kidney disease (50), diabetic cardiomyopathy (51) and diabetic neuropathy (52), which is further elucidated below. In fact, Nrf2 has been indicated to be involved in mediating all aspects of diabetic complications across every diabetes-relevant organ (43).

The Nrf2 activators up-regulate the expression of Sestrin2 in a time- and dose-dependent manner and the Nfr2-ARE pathway activation seems to be necessary for Sestrin2 induction. In turn, Sestrin2 can function as a positive regulator of Nrf2 signaling, activate the Nrf2 pathway by augmenting autophagy-directed degradation of Keap1, which targets and breaks down Nrf2 (6, 53). Sestrin2 overexpression suppresses oxidative stress and cell apoptosis by activating Nrf2-ARE signaling (54, 55). Also, Sestrin2/Nrf2 signaling may be important for the mediation of ER stress as a downstream regulator of the protein kinase R-like endoplasmic reticulum kinase (PERK) pathway (56), which is illustrated below.

### 2.3 Sestrin2 and autophagy

Autophagy is a distinct type of programmed cellular death. Autophagy helps maintain cell survival and tissue stabilization by degrading misfolded and aged intracellular proteins and dysfunctional organelles during stress. The process of autophagy is regulated by various pathways and involves diverse organelles such as mitochondria, ER, ribosomes, peroxisomes, and lysosomes. The dysfunction of autophagy is related to a myriad of diseases, such as diabetes, cardiovascular diseases, cancer, neurodegenerative diseases, liver diseases, and inflammatory diseases (57–59). Autophagy takes part in the regulation of pancreatic β-cells and protection of insulin target tissues. Dysfunctional autophagy is detrimental for the maintenance of β-cell function and reduces insulin secretion. Furthermore, inhibition of autophagy leads to chronic ER stress and β-cell apoptosis. The disruption of autophagy also contributes to diverse diabetic complications (1, 60).

Autophagy activation is required for the antioxidant effects of Sestrin2 (61). After activated by the JNK pathways (62), Sestrin2 is involved in modulation of autophagy through AMPK/mTORC1, Keap1/Nrf2, p53/Sestrin2 and PI3K/AKT/mTOR pathways (63–65). Furthermore, Sestrin2 has been indicated to be interacted with BCL2/adenovirus E1B 19 kDa protein-interacting protein 3 (BNIP3), which is also a promoter of autophagy (66).

### 2.4 Sestrin2 and oxidative stress

Oxidative stress is considered to be an imbalance in redox properties in certain cellular environments, and plays a crucial role in the development of numerous human diseases, such as diabetes, obesity, and myocardial injury (67, 68). Oxidative stress has been proved to play key roles in the pathogenesis of diabetes and diabetic complications. The high glucose activates various molecular and biochemical pathways, causing increased ROS production, which thereby leads to insulin resistance, β-cell dysfunction and diabetic complications (69).

Sestrin2 is essential for the maintenance of cellular homeostasis under oxidative stress. In various types of diseases, Sestrin2 is up-regulated and is important for the resistance to oxidative stress injury. Under oxidative stress, Sestrin2 is activated by various transcription factors, including NF-κB, activator protein-1 (AP-1), CCAAT-enhancer-binding protein beta (C/ERPβ), forkhead box O3 (FOXO3), and p53 (70). Sestrin2 helps to maintain the balance of oxidative metabolism by exerting two major biological functions. First, as an antioxidant enzyme, Sestrin2 is able to directly reduce the accumulation of ROS (71). Second, Sestrin2 can exert antioxidant effects through several signaling pathways, such as Keap1/Nrf2 pathway (6) and AMPK/mTORC1 pathway (72), which have been described above.

### 2.5 Sestrin2 and ER stress

ER stress is provoked when unfolded or misfolded proteins accumulate in the endoplasmic network lumen in pathophysiological conditions (73). Many physiological and pathological factors, such as inflammation, hypoxia and oxidative stress, disturb the homeostasis of ER and lead to ER malfunction, which thereby causes ER stress and promotes the unfolded protein response (UPR). Three ER transmembrane receptors inositol-requiring enzyme 1 (IRE1, also known as ERN1), PERK and activating transcription factor 6 (ATF6) mediate ER state by regulating UPR (74). The activation of UPR impairs cellular survival by improving protein folding ability, inhibiting protein production and accumulation, inducing ER stress-related gene transcription, and strengthening the self-repair ability of ER. But if ER stress persists or continues for prolong periods, UPR is not enough to maintain ER homeostasis, and apoptosis ultimately occurs (75). ER stress has significant impact on maintaining cellular homeostasis (76). ER stress plays mediating roles in the pathogenesis of a series of diseases, such as diabetes, obesity, inflammation, neurodegenerative diseases, cancer, and autoimmune diseases (77). Numerous studies have proved the role of ER stress in diabetes. Disturbed ER homeostasis and unmitigated ER stress trigger or exacerbate β-cell dysfunction, and contribute to insulin resistance in diabetes (60, 78). Diabetic complications are closely associated with dysregulation of UPR signaling pathways (79).

Increasing evidence has shown that Sestrin2 is activated under ER stress (8, 80). How Sestrin2 expression is induced by ER stress is not fully revealed. The PERK and IRE1/X-box binding protein 1 (XBP1) arms of the UPR appear to be required (8). Also, the activation of transcription molecules, such as HIF-1, activating transcription factor 4 (ATF4) and Nrf2, is suggested to be necessary for ER stress-induced expression of Sestrin2 (72). Once induced, Sestrin2 in turn prevents protein synthesis by inhibiting mTORC1 (81). Sestrin2 inhibits the phosphorylation of JNK and p38 as well as poly ADP-ribose polymerase (PARP) cleavage, and prevents the adverse effect of excessive ER stress (80). The AMPK/mTORC1 pathway, Keap1/Nrf2 pathway, CCAAT-enhancer-binding protein homologous protein, phosphorylation of both p38 and JNK, and Sestrin2-mediated UPR is involved in the protective effects of Sestrin2 against ER stress-associated diseases (4, 82, 83).

### 2.6 Sestrin2 and mitochondrial function

Mitochondria are the prime organelle which not only offers energy substrates to cells but also controls the fate of cells via mediating diverse cellular processes such as autophagy, apoptosis, cellular mobilization and metabolism (84, 85). Mitochondria possess a quality control system, including mitochondrial dynamics (fusion and fission), mitophagy and mitochondrial biogenesis, which is critical for maintaining a well-functioning mitochondrial network (86, 87). Altered mitochondrial functionality is involved in a variety of diseases, such as diabetes, obesity, neurological disorders, and cardiovascular diseases (88–91). A myriad of evidence has revealed crucial roles of mitochondrial dynamics, mitophagy, and mitochondrial biogenesis in the pathogenesis of diabetes. Dysregulations of mitochondrial functions and dynamics could result in β-cell dysfunction and insulin resistance (92, 93).

Recent studies have showed that Sestrin2 may play an important role in maintaining cellular homeostasis by restoring mitochondrial function and metabolism (7, 94). Mitochondrial superoxide mediates Sestrin2 activation in the process of mitochondrial quality control (95). Sestrin2 can thereby secure the mitochondria from oxidative lesion, both *in vivo* and *in vitro* (96, 97). Mitophagy is a subtype of autophagy, which helps to remove dysfunctional mitochondria as well as is crucial for maintenance of the functionality and integrity of the mitochondrial network. Several studies indicated that Sestrin2 is involved in regulating the pace of mitophagy (95, 98). Sestrin2 stimulates ULK1- mediated phosphorylation of Beclin1 and strengthens the interaction between Beclin1 and Parkin. Then mitophagy is provoked as Parkin’s shift on the surface of mitochondria (95, 99). Sestrin2-mediated autophagy and mitophagy can ameliorate mitochondrial dysfunction and prevent cell apoptosis (100).

Several signaling pathways participate the regulating mechanisms of Sestrin2 in mitochondrial function and metabolism. Kim et al. reported that Sestrin2 suppresses the overactivation of the NLRP3 inflammasome and alleviates mitochondrial injury. Sestrin2 promotes perinuclear clustering-damaged mitochondria through regulating the aggregation of SQSTM1 and its binding to Lys63-linked ubiquitins on the surface of damaged mitochondria (98). Sestrin2 overexpression suppresses inflammation by inducing AMPK/PGC-1α-mediated mitochondrial biogenesis (101). Sestrin2/LKB1/AMPK pathway is also indicated to function in mitochondrial quality control enhancement, including mitochondrial biogenesis and mitophagy (38).

### 2.7 Sestrin2 and apoptosis

Apoptosis is an active programmed cell death process, characterized by specific biochemical and morphological alterations such as cellular shrinkage, nuclear condensation and chromatin condensation along the nuclear membrane (102). There are three major signaling pathways that modulate apoptosis, namely the mitochondrial, death receptor and ER pathways (103). Pancreatic β-cell apoptosis is the determining factor for the decline of β-cell function and impaired insulin secretion in diabetes (104). Also, apoptosis of organ-specific cells has been identified and characterized in the development of diabetic complications (105, 106).

In different cell types and under different pathophysiological conditions, Sestrin2 may exert opposite effects on apoptosis. In most studies targeting non-tumor cells, Sestrin2 is involved in anti-apoptotic signaling pathways. However, in most tumor studies, Sestrin2 elicits proapototic effects in cancer cells (107). Ding B et al. reported that Sestrin2 is protective for overall cell energy metabolism and mitochondrial function. Sestrin2 overexpression can reduce cell apoptosis by reducing ROS aggregation, maintaining mitochondrial membrane potential, reducing ATP consumption and restoring mitochondrial DNA level (7). However, as shown by the study of Seo K et al. (108) and Budanov AV et al. (109), Sestrin2 overexpression can promote cell apoptosis. Bidirectional regulating roles of Sestrin2 in apoptosis are indicated and require further validation (110).

## 3 The roles of Sestrin2 in diabetes

**Figure 1:**
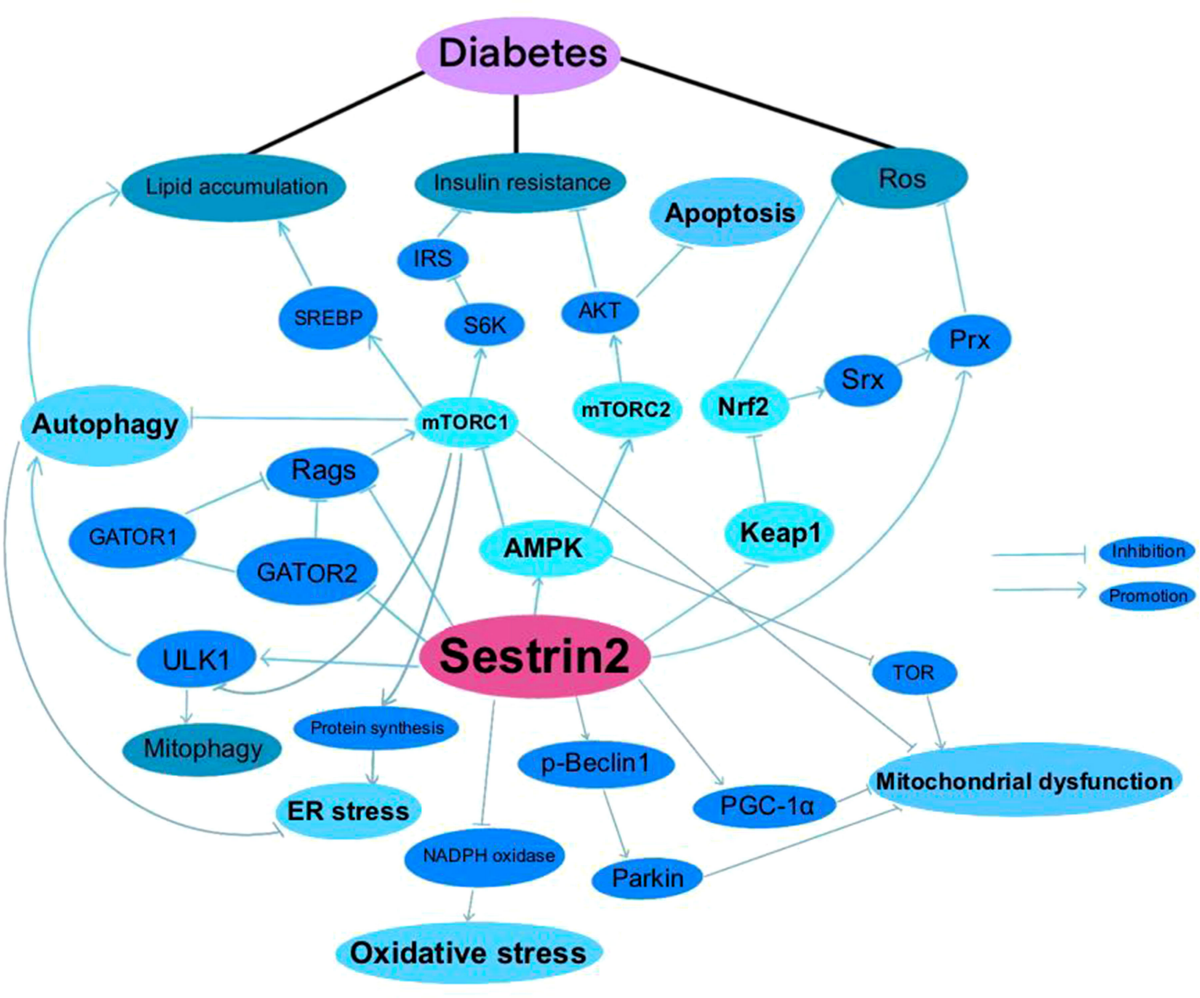
Sestrin2 and Diabetes.

A summary of researches which investigated the roles of Sestrin2 in diabetes, diabetic complications and diabetes-associated conditions is presented in **Table 1**. **Figure 1** summarizes the effects of Sestrin2 on diabetes-associated signaling pathways. As mentioned before, diabetes is characterized by changes in AMPK and mTOR, the principle nutrient level sensing mechanisms (129). The chronic continuous activation of mTORC1 is accompanied by continuous inhibition of hepatocyte autophagy, leading to insulin resistance and T2DM mainly by suppressing the phosphorylation of insulin receptor substrates (130). Continuous cellular mTORC1 activation under overnutrition promotes protein and lipid synthesis and inhibits autophagy catabolism (111). One of the major negative feedback mechanisms that prevent the harmful effects of chronic mTORC1 continuous activation is the transcriptional activation of Sestrin2. Chronic mTORC1 activation mediated by stress responses such as overnutrition eventually results in Sestrin2 overactivation (111, 131). After activation, Sestrin2 stimulates AMPK signaling, which in turn impairs mTORC1 activation and, therefore, triggers autophagy (111, 132). The major target organs and tissues of insulin resistance include liver, muscle and adipose tissue. Sestrin2 is found to be highly accumulated in muscle, liver, and adipose tissues in models of T2DM and obesity (41, 111). As reported by Lee et al., Sestrin2 can activate AMPK, attenuate mTORC1-S6K activity in the liver, thereby lowering blood glucose level in obese mice. Sestrin2 ablation activates hepatic mTORC1-S6K signaling, and enhances insulin resistance, hepatic steatosis and diabetic progression, indicating a key role of Sestrin2 in cell metabolic homeostasis (111). Insulin up-regulates Sestrin2 expression in mouse primary hepatic cells and the upregulation of Sestrin2 by insulin was shown to be regulated via PI3K/PKB/mTOR signaling pathway, indicating a feedback mediation of Sestrin2 on insulin signaling transduction (112). Also, Sestrin2 is identified to induce autophagy, maintain insulin sensitivity and glucose metabolism by regulating AMPK/mTORC1 signaling pathway (64, 113). Sestrin2/AMPK/mTORC1 signaling pathway is indicated to contribute to the maintenance of β-cell function and resistance to pathological stresses associated with diabetes (114). Therefore, based on these evidences, Sestrin2 is a potential insulin sensitizer and one of the key factors for β-cell homeostasis. Deficiency and/or dysfunction of Sestrin2 may result in insulin resistance and the development of diabetes (70).

**Table 1:** The roles of Sestrin2 in diabetes and diabetic complications.

Type of disease/intervention	Model	Pathway	Effect	Reference
Sestrin2 in diabetes associated conditions				
Obesity	Mice	Sestrin activates AMPK, suppresses mTORC1-S6K.	Maintains metabolic homeostasis.	(111)
Insulin signaling	Primary hepatic cells	Insulin upregulates Sestrin2 through PI3K/PKB/mTOR.	Negative feedback effect on insulin signaling.	(112)
Exercise	Mice	Sestrin2 interacts with AMPK and activates autophagy.	Mediates the effects of exercise to increase insulin sensitivity.	(113)
Insulin resistance	C2C12 myotubes	Sestrin2 induces autophagy via activation of AMPK.	Maintains insulin sensitivity and glucose sensitivity.	(64)
Pathological stresses relevant to diabetes	Pancreatic β-cells	Sestrin2 promotes autophagy by attenuating mTORC1 activaiton through AMPK-dependent and -independent mechanisms.	Maintains β-cell function.	(114)
Regular or obesity	Interscapular brown adipose tissue in mice	Sestrin2 suppresses UCP1 expression.	Regulates thermogenesis and mitohormesis. Either overexpression or deficiency of Sestrin2 is detrimental for the energy homeostasis in brown adipose tissue.	(115)
β3AR agonist	Inguinal white adipose tissue and soleus muscle in mice	Sestrin2 regulates β3AR.	Reduces lipid droplet size in inguinal white adipose tissue and increases soleus muscle mass.	(56)
High-glucose and dyslipidemic conditions	Monocyte	Sestrin2 regulates AMPK/mTOR signaling and also AMPK regulates Sestrin2 in a feedback mechanism.	Regulates monocyte activation.	(116)
High-fat diet-induced obesity	Mice	Sestrin2 activates AKT through GATOR2-mTORC2 axis.	Promotes metabolic homeostasis.	(41)
Obesity with T2DM	Obesity children with T2DM		Serum Sestrin2 is decreased.	(117)
T2DM	Patients with T2DM		Serum Sestrin2 is decreased.	(118)
T2DM	Patients with T2DM		Serum Sestrin2 is decreased.	(119)
T2DM	Newly diagnosed drug-naïve T2DM		Serum Sestrin2 is increased.	(120)
T2DM	Patients with T2DM		Serum Sestrin2 is increased.	(121)
Sestrin2 in diabetic complications				
Diabetic kidney disease (DKD)				
Diabetic kidney disease (DKD)	Human samples and podocytes and diabetic rats	Sestrin2 regulates AMPK in mitochondria and podocytes.	Sestrin2 expression is decreased in podocytes from patients with DKD and in podocytes from diabetic rats. Sestrin2 circumvents mitochondrial dysfunction.	(122)
Diabetic nephropathy	T2DM patients with diabetic nephropathy		Serum Sestrin2 is decreased, especially in those with macroalbuminuria.	(123)
Diabetic kidney disease (DKD)	HK-2 cells	MIR-4756 suppresses Sestrin2.	Promotes albumin-induced renal tubular epithelial-to-mesenchymal transition and endoplasmic reticulum stress.	(124)
Diabetic kidney disease (DKD)	Diabetic mice, mouse podocytes exposed to high glucose	Sestrin2 modulates TSP-1/TGF-β1/Smad3 pathway.	Mitigates podocyte injury in DKD.	(22)
Diabetic kidney disease (DKD)	Diabetic mice	Sestrin2 regulates Hippo pathway.	Inhibits mesangial cell proliferation and fibrosis.	(125)
Diabetic heart disease (DHD)				
Coronary heart disease (CHD)	T2DM patients with CHD		Serum Sestrin2 is decreased, and is a risk factor for CHD.	(126)
Diabetic myocardial ischemia/reperfusion injury	Diabetic rat	Sestrin2 interacts with Nrf2.	Promotes antioxidant actions and attenuates mitochondrial damage.	(18)
Diabetic cardiomyopathy	Cardiomyocytes exposed to high glucose		Alleviates mitochondrial dysfunction and ameliorates cardiac injury.	(127)
Diabetic neuropathy (DN)				
Diabetic neuropathy (DN)	T2DM patients with diabetic peripheral neuropathy		Serum Sestrin2 is decreased.	(128)

Besides the role in the modulation of insulin signaling, there are studies referring to the regulating effects of Sestrin2 on peripheral tissues which play key roles in the pathogenesis of diabetes, such as adipose tissues and skeletal muscle. Growing evidence displays adipose tissue as an endocrine organ which produces multiple adipokines regulating diverse aspects of β-cell function and viability. Adipose tissue malfunction is crucially involved in the development of diabetes (133, 134). Recent finding suggested Sestrin2 as a regulator of motohormesis in brown adipose tissue (135). Also, Sestrin2 was found to be related to beneficial body composition changes, including the decrease of lipid droplet size in inguinal white adipose tissue and the increase of soleus muscle mass (56). Monocytes and macrophages are critically involved in atherosclerosis and participate in the atherosclerotic lesion progression associated with diabetes (136). Sestrin2 was shown to play a principal role in regulating monocyte activation through the AMPK/mTOR pathway in diabetes and also AMPK mediates Sestrin2 in a feedback way (116).

As described before, except for the classical AMPK/mTORC1 pathway, Sestrin2 can exert downregulating effects on mTORC1 through other mechanisms such as GATOR2-GATOR1-mTORC1 signaling pathway (39). On the other hand, Sestrin2 could increase mTORC2 activity through its ability to interact with mTORC2 via GATOR2-mTORC2 signaling pathway during high-fat diet-induced obesity (41). However, these mediating pathways of Sestrin2 on mTORC have not been investigated and confirmed in diabetic models.

Several clinical studies have investigated the changes of circulatory levels of Sestrin2 in patients with obesity, T2DM, and metabolic syndrome. However, no consensus was reached. It was indicated that circulatory Sestrin2 is lowered in diabetes and negatively correlates with glycemic levels (117, 118, 123). Also, as shown by the study of Golpour et al., plasma Sestrin2 level presents a trend of decrease in obesity and T2DM (119). On the contrary, some studies reported significant high serum levels of Sestrin2 in patients with T2DM, obesity, and metabolic syndrome (120, 121). Sestrin2 concentration significantly correlates with insulin resistance and percentage body fat (120).

## 4 The roles of Sestrin2 in diabetic complications

Rather than a disease of mere hyperglycemia, diabetes brings real harm and devastating effects by leading to a series of complications in peripheral systems, organs and tissues such as kidney, cardiovascular system, retina, and the nervous system (137). Diabetic complications are often irreversible, causing severe injury and increasing mortality in patients with diabetes. Recent researches have suggested the contribution of alterations of Sestrin2 and the related pathways in the pathogenesis of diabetic complications.

### 4.1 Diabetic kidney disease

Diabetic kidney disease (DKD) is a typical chronic microvascular diabetic complication and is a major cause of chronic kidney disease and end-stage renal disease worldwide (138). About 30%-50% of patients with T1DM or T2DM eventually develop DKD, resulting a significant increase of mortality in these patients. Clinically, patients with DKD often exhibit proteinuria, hypertension and edema, while laboratory tests present increased urinary albumin excretion and decreased estimated glomerular filtration rate (eGFR). A series of signaling pathways contributes to the pathogenesis of DKD, including AMPK/mTOR pathway, MAPKs/Erk1/2 pathway, PI3K/AKT pathway and the advanced glycation end products (AGEs) pathway (139–141). As shown by Puelles et al., hyperglycemia can induce oxidative stress and other pathophysiological processes through AMPK/mTOR signaling, leading to podocyte injury and proteinuria, therefore leading to the loss of renal function (139). Activated mTORC1 signaling is a feature of DKD, which causes podocyte and tubular damage by suppressing autophagy and in turn promotes progressive kidney dysfunction (142, 143).

The activation of Sestrin2 could inhibit AMPK/mTOR signaling, promote autophagy and reduce the susceptibility of renal cells to diabetes-related damage. The potential therapeutic role of Sestrin2 in DKD was initially found in a human proximal tubule cell line (HK-2) model, illustrating that overexpression of Sestrin2 represses DKD-induced renal epithelial tubular cell epithelial-to-mesenchymal transition and ER stress, but its mechanism is still unclear. The researchers further found that administration of microRNA-4756 regulates DKD-induced renal tubular epithelial cell damage by the interaction with Sestrin2 (124). Later, Lin et al. reported that Sestrin2 activation increases the level of AMPK phosphorylation, and thereby ameliorates mitochondrial dysfunction of podocytes under high glucose conditions (122). However, it is worth noting that though overactivation of mTORC1 in diabetes aggravates kidney lesions, mTOR activity is necessary to maintain podocyte homeostasis. Genetic deletion of mTOR in mouse podocytes induces proteinuria and progressive glomerulosclerosis. A tightly balanced mTOR activity is essential to maintain normal renal function in diabetes (142). Clinically, serum Sestrin2 levels were found to significantly decrease in T2DM patients with diabetic nephropathy, especially in the ones with macroalbuminuria (123). In recent years, sodium-glucose co-transporter 2 (SGLT2) inhibitors have been well-documented to protect the renal function in patients with and without T2DM and slow down the progression towards end-stage kidney disease (144). It has been shown that the Sestrin2/AMPK pathway plays a critical role in the protective actions of SGLT2 inhibitors on metabolism, fibrosis, and organ damage in obese mice (145). Specially, studies have demonstrated that activation of AMPK by inhibiting SGLT2 is a main protective mechanism in diabetic nephropathy (146). Nevertheless, in another study investigating the working mechanisms of empagliflozin, Sestrin2/AMPK pathway was not activated in nondiabetic rats and did not participate in the renal protective effects of empagliflozin (147).

TGF-β1 is a decisive regulator of renal fibrosis and overactivation of TGF-β1 could cause progressive renal injury (148). Hyperglycemia and insulin resistance enhance the expression of Angiotensin II, which increases ROS production and activates TGF-β1 signaling (149). Smad2/3 complex, PI3K/AKT/mTOR, protein kinase C (PKC), MAPK, interleukin like kinase (ILK) and Wnt/beta-catenin signaling are among the downstream targets that modulate profibrogenic effects of TGF-β1 (150–152). Thrombospondin-1 (TSP-1) is an extracellular matrix protein that mediates a wide range of biological processes. TSP-1 is vital to maintain normal glucose metabolism. Also, TSP-1 is involved in the pathophysiology of multiple diabetic complications, including diabetic cardiomyopathy, neuropathy and nephropathy (153). TSP-1 mediates the activation of latent TGF-β1, which is indispensable for maintaining the normal function of islet. Nevertheless, during chronic hyperglycemia, TGF-β1 exacerbates diabetic nephropathy by inducing renal fibrosis (154). Both TGF-β and TSP-1 have been indicated to play causal roles in insulin resistance and obesity-related renal fibrosis, except for TGF-β-dependent and independent roles of TSP-1 (155, 156). Recently, Song et al. reported that Sestrin2 remedies podocyte injury in DKD through the coordination with TSP-1/TGF-β1/Smad3 pathway, suggesting that Sestrin2/TSP-1/TGF-β1 signaling is critically involved in renal protection (22).

**Figure 2:**
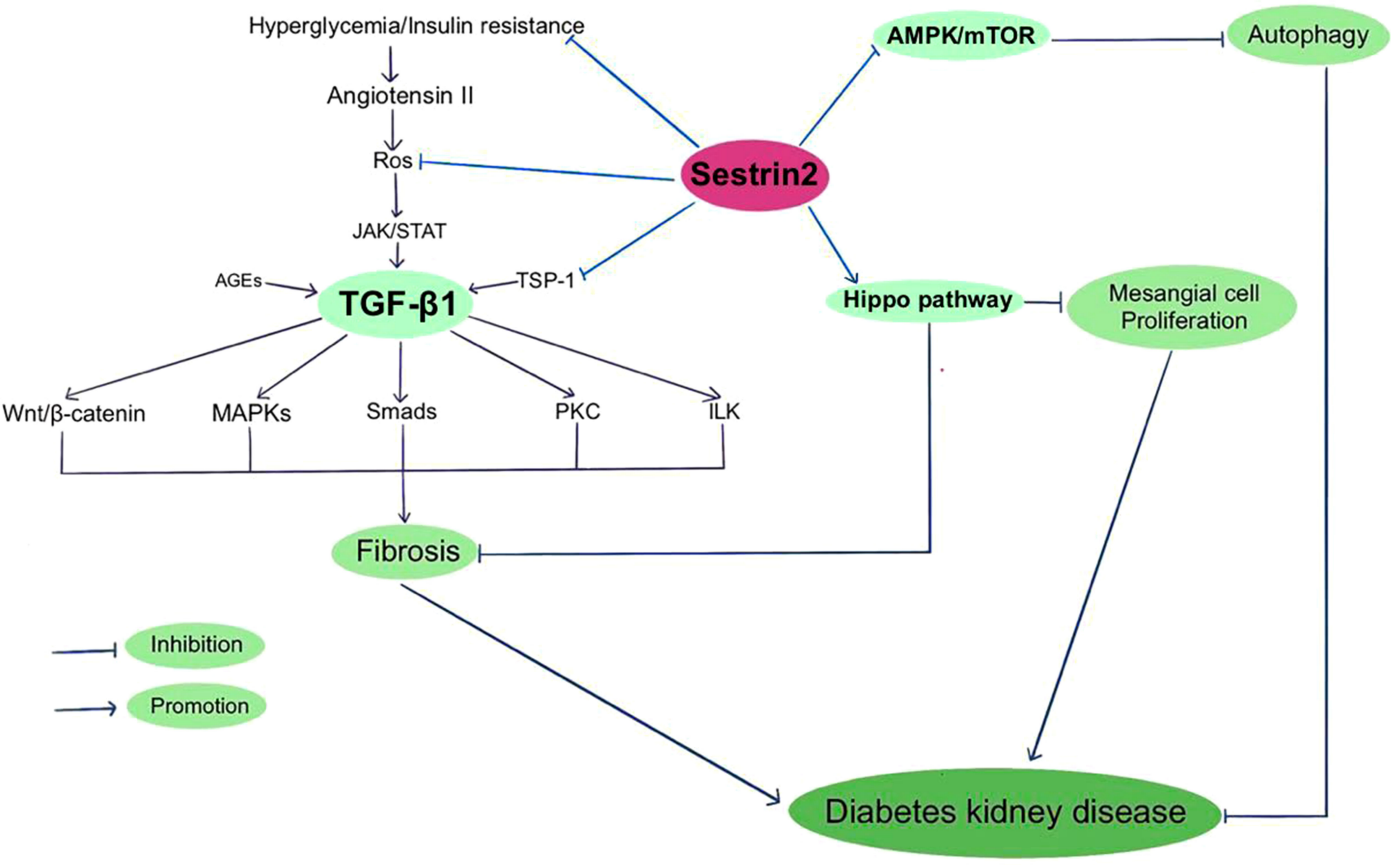
Sestrin2 and Diabetic kidney disease.

The Hippo pathway, a kinase cascade that regulates cellular proliferation, differentiation, and tissue homeostasis, is inhibited in diabetic conditions. The Hippo pathway has been indicated to be involved in the development and progression of DKD (157, 158). PI3K/AKT signaling is related to the Hippo pathway and both of these pathways take part in the pathogenesis of DKD (158). Sestrin2 overexpression was found to alleviate renal damage via regulating Hippo pathway in DKD mice (125). Considering the interactions among Sestrin2 and multiple signaling pathways, Sestrin2 may be critically in involved in the development of DKD and thus may perform as a latent therapeutic target for DKD. **Figure 2** summarizes Sestrin2 signaling pathways in diabetic kidney disease.

### 4.2 Diabetic cardiovascular complications/diabetic heart disease

Diabetic heart disease (DHD) is a major cause of death in patients with diabetes. It refers to abnormal heart structure and manifestations in patients with diabetes in the absence of other cardiac risk factors. DHD is a conglomeration of coronary artery disease, heart failure, diabetic cardiomyopathy (DCM) and diabetic cardiac autonomic neuropathy, and is characterized by molecular, structural, and functional changes in the myocardium (159, 160). The pathogenesis includes the macrovascular and microvascular lesions and cardiac autonomic neuropathy caused by oxidative stress, inflammatory response, mitochondrial dysfunction, AGEs, alterations at the level of insulin signaling, gene regulation, ER stress, hypoxia, neurohumoral activation, apoptosis, and exosome dysregulation (160, 161).

mTOR signaling is found to play a key role in the development of DHD. Activation of mTORC1 either by strengthening PI3K/AKT signaling or disruption of tuberous sclerosis complex 1 (TSC1) drives cardiac hypertrophy (162, 163). Also, mTORC1 inhibition exerts cardioprotective effect against myocardial ischemia and DCM by activating autophagy (164). Inhibition of mTOR signaling by application of melatonin reduces myocardial damages and protects against DCM (165). mTORC2 seems to exert reverse effect on cardiac remodeling. Suppression of mTORC1 and activation of mTORC2 exert beneficial effects on myocardial ischemia and adverse cardiac remodeling (29). TGF-βs are central effectors of myocardial fibrosis (166). TGF-β-driven fibrosis is regulated by canonical or noncanonical pathways and is mediated by coreceptors and by interacting networks. The activation of canonical or Smad-dependent signaling pathways causes phosphorylation and activation of SMAD proteins. The activation of noncanonical pathways include PI3K/AKT, ERK, JNK, RhoA and MAPK pathways (167, 168). In the dilated cardiomyopathy model, the increase of myocardial expression of TGF-β and activation of downstream Smad 2 and Smad 3 signal cascades have been unanimously confirmed. Overexpression and activation of TGF-β1 in DCM induces cardiac fibrosis, which can be alleviated by administration of telmisartan, empagliflozin, dapagliflozin, **e**pigallocatechin **g**allate, or cannabidiol, possibly due to the inhibitory effects on TGF-β signaling (169–174). TSP-1 is suggested to play a significant role in DCM. TSP-1 upregulation in the diabetic heart stabilizes the cardiac matrix and promotes vascular rarefaction in obese diabetic mice. TSP-1 enhancement in the myocardium may be a crucial regulator in diabetes-associated impaired angiogenesis (175). The effect of TSP-1 are mediated regulated by activation of TGF-β, angiostatic actions, matrix metalloproteinase inhibition and direct stimulation of CD36 signaling (176). Downregulating TSP-1 and TGF-β1 improves the heart function and ameliorates vascular fibrosis in diabetic rats (177, 178).

**Figure 3:**
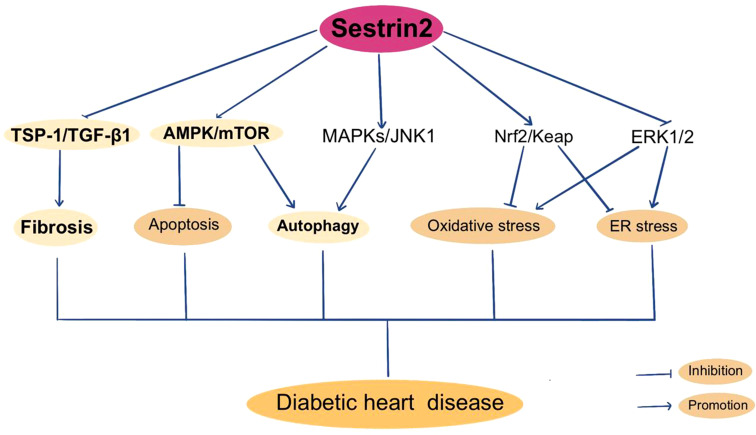
Sestrin2 and Diabetic heart disease.

As previously described, Sestrin2 participates in the modulation of oxidative stress, mitochondrial biogenesis, ER stress and apoptosis. Also, the AMPK/mTOR pathway and TSP-1/TGF-β1 pathway are constitute parts of the regulating mechanism of Sestrin2. It is reasonable to postulate that Sestrin2 may take part in the pathogenesis of DHD. But the researches investigating the role of Sestrin2 in DHD are relatively few *(rare)*. Previously, Sestrin2 is considered to be cardioprotective in several models of cardiovascular diseases, including myocardial infarction and cardiac dysfunction induced by ER stress or lipopolysaccharide, via AMPK/mTOR signaling cascade (97, 179). Besides, increasing evidence indicate a protective role for Sestrin2 against the development and progression of cardiomyopathy and heart failure in model of pressure-overload cardiac remodeling, via Nrf2/Keap1 pathway (180). Also, Sestrin2 is indicated to modulate cardiac inflammatory response through maintaining oxidative stress through MAPK/JNK pathway during ischemia and reperfusion (181). Sestrin2 suppression aggravates ER stress-induced oxidative stress and apoptosis in endothelial cells (182). Clinically, several studies have investigated the variations in plasma Sestrin2 protein levels in patients with cardiomyopathy and/or heart failure, and displayed conflicting results (183–185). Wang et al. reported that plasma Sestrin2 level was increased in patients with chronic heart failure (CHD) and was positively related to the severity of CHD. Increment of Sestrin2 concentrations prominently increased the occurrence of major adverse cardiac events and suggested poor prognosis (183). Also, plasma Sestrin2 levels were found to be increased in patients with coronary heart disease (CAD) and positively related to the severity of CAD (184, 186). However, lower serum Setrin2 levels were indicated in patients with septic cardiomyopathy and in T2DM patients with CHD (126, 185). Low Sestrin2 level was a risk factor for CHD in T2DM patients (126). The contradictory results concerning the beneficial or harmful effects of sestrin2 in cardiomyopathy and heart failure need to be further clarified. In recent years, a few studies investigated the role of Sestrin2 in DHD. Zhou et al. reported that Sestrin2 may enhance antioxidative actions and alleviate mitochondrial lesion by interacting with Nrf2 to prevent the diabetic rat heart from ischemia/reperfusion injury (18). Our previous research showed that inhibition of enhanced Sestrin2 expression attenuates cardiac injury in DCM, which may be mainly attributed to the restoration of mitochondrial function (127). Some antiglycemic agents, such as metformin and empagliflozin, were found to be cardioprotective through Sestrin2-associated mechanism. As shown by Yang et al., metformin can activate AMPK, thereby promoting autophagy by suppressing the mTOR pathway in DCM (187). Sestrin2 was suggested to participate in cardioprotective effects of metformin in a model of acute kidney injury (188). Sun et al. found that empagliflozin improves obesity-related cardiac dysfunction via regulating Sestrin2-mediated AMPK/mTOR signaling and maintaining redox homeostasis (145). **Figure 3** summarizes Sestrin2 signaling pathways in diabetic heart disease.

### 4.3 Diabetic ocular complications

Diabetes can cause various ocular complications, such as diabetic retinopathy (DR), cataract, diabetic papillopathy, glaucoma, and ocular surface diseases (189). DR is a major diabetic complication characterized by retinal microvascular lesion and is a major cause of vision loss in working middle-aged adults. Complicated mechanism is included in the pathogenesis of DR, including increased free radical production, activated AMPK/mTOR signaling, renin-angiotensin pathway, TGF-β/Smad signaling, and the kallikrein-kinin system, the formation of AGEs, and increased inflammatory factors and vascular endothelial growth factor (VEGF) (190–192). mTOR signaling is considered to play multiple roles in the pathogenesis of DR. mTORC1 is indispensable for the hypoxic-induced expression of VEGF, which is an important pathogenic event in DR (193). Also, mTORC1 affects DR development by negatively regulating autophagy (194). PI3K/AKT/mTOR signaling pathway is associated with the early pathogenesis of DR (195). Promoting autophagy and enhancing AMPK/mTOR signaling pathway can protect retinal Muller cells from apoptosis caused by high glucose (196). Aberrant TGF-β signaling pathway is involved in the pathogenesis of DR (155). TGF-β1 protects retinal ganglion cells from hyperglycemia-induced oxidative damage through promoting cell antioxidation and neuroprotection pathways, including Nrf2/Keap1 signaling (197). Increased TGF-β signaling induced by diabetes protects retinal vessels in diabetic rats and may prevent rapid retinopathy progression (198).

Based on its pleiotropic modulating effects, Sestrin2 may have an impact on the pathogenesis of DR. So far there are several studies exploring the role of Sestrin2 in models of ocular lesions, but still no reports in diabetic ocular complications have been found. Previously, Hanus et al. demonstrated that upregulation of Sestrin2 protects retinal pigment epithelial cells from oxidative stress-induced necrosis (199). Sestrin2 could also secure retinal ganglion cells from oxidative stress-induced apoptosis through Keap1/Nrf2 pathway, which suggests a significant role of Sestrin2 in retinal degeneration in glaucoma (200). But on the other hand, Sestrin2 is indicated to be a negative modulator of corneal epithelial cell proliferation. The downregulation of Sestrin2 leads to the synergistic activation of mTORC1 and Hippo signaling, thus promoting reepithelialization of the corneal wound (201).

### 4.4 Diabetic neuropathy

Diabetic neuropathy (DN) is another frequent chronic complication of diabetes, consisting of four major types including peripheral neuropathy, autonomic neuropathy, proximal neuropathy, and mononeuropathy (202). The pathogenesis of DN is complicated, including changes of various metabolic pathways and vascular pathways. Three main pathological events contribute to the progression of DN, including chronic low-grade inflammation, endothelial dysfunction and oxidative stress (203). Consistent hyperglycemia in diabetes induces activation/inhibition of diverse pathways, including polyol, hexosamine, AGEs, PARP, MAPK, mTOR, NF-κB and tumor necrosis factor-α (TNF-α) pathway, which contribute to the pathogenesis and progression of DN (204, 205). Among them, overactive mTORC1 interferes with synaptic plasticity and is one of the main factors contribute to chronic neuropathy. Activation of mTOR exacerbates the hyperalgesia in diabetic rats, while suppression of mTORC1 activity is indicated to lead to an anti-injury effect in experimental model of diabetic small fiber neuropathy (206, 207). With the inhibition of PI3K/AKT/mTOR pathway, autophagy is enhanced and hyperalgesia is alleviated in diabetes rats (208). So far, no research exploring the role of Sestrin2 in DN can be found. But there are a few studies in other disease models. In denervated atrophy, Sestrin2 has been proved to prevent the change of muscle fiber type from slow contraction to fast contraction through AMPK/PGC-1α signaling, and protect muscle quality (209). Regulation of UPR and mitophagy is also included in the mechanism by which Sestrin2 protects against denervated muscle atrophy (210). Zhang et al. demonstrated that overexpressing Sestrin2 significantly reduces oxidative stress of neurons in model of cerebral ischemia-reperfusion injury through modulating the activity of Nrf2 (12). Mao et al. conducted a clinical study and documented that serum Sestrin2 is lowered in T2DM patients with diabetic peripheral neuropathy (128).

## 5 Possible pharmacological mediators of Sestrin2

The exploration of Therapeutic strategies through mediation of Sestrin2 is now underway. Several natural products and medications in diabetes have been shown to alter the expression levels of Sestrin2, which lead to the possibility of novel treatments in diabetes and diabetic complications targeting Sestrin2 and the associated pathways (211). Initially, a few studies investigated the potential mediator of Sestrin2 in the field of tumor therapies, including several small molecules (212). Recently, natural-derived mediator of Sestrin2, such as Gallic acid (213) and eupatilin (Unpublished data of our research), are also indicated to be potential therapeutic agents of obesity and diabetes. Some antidiabetic medications are indicated to be involved in the regulation of Sestrin2 signaling in diabetes-associated conditions, some of which have been illustrated in the previous sections of this review. Sestrin2 can be targeted by empagliflozin in the treatment of obesity-related nonalcoholic fatty liver disease (214). Another antidiabetic agent, liraglutide, is shown to alleviate obesity-related fatty liver disease via modulating the Sestrin2-mediated Nrf2-HO-1 pathway (215). Intervention through gene editing of Sestrin2 also presents beneficial effects in organ and tissue protection (216), but further investigation is needed in the context of diabetes.

## 6 Conclusions and perspectives

Diabetes is a condition causing multi-organ injuries and is a major global threaten for human health. As a stress-induced protein, Sestrin2 can be activated by diverse stresses and can exert pleiotropic effects. Sestrin2 can interact with various signaling perspectives involved in the development of diabetes. Increasing numbers of studies indicate a prominent role of Sestrin2 in the pathogenesis of diabetes and diabetic complications. Sestrin2 may functions in a multitude of ways and offer exciting prospects for the treatment of diabetes and diabetic complications, though currently the strong supporting evidence is limited. Despite of the protective roles of Sestrn2 in various conditions, the pros and cons of excessive activation or inhibition of Sestrin2 is yet to be confirmed. How to exert accurate mediation under different conditions also remains elusive. The modulation of Sestrin2 activity to effectively achieve homeostasis might be more appropriate. Further researches are needed to thoroughly reveal the relationship between Sestrin2 and diabetes which includes multi-organ injuries, to disclose associated signaling pathways and to explore potential treatment protocols.

## Author contributions

XZ: Writing – review & editing, Conceptualization, Writing – original draft. ZL: Writing – original draft. JL: Writing – original draft. YLin: Writing – original draft. YLi: Writing – original draft, Project administration, Resources, Software. WL: Writing – review & editing, Supervision, Validation.
